# Glucose-6-Phosphate Dehydrogenase Deficiency and Neonatal Hyperbilirubinemia: Insights on Pathophysiology, Diagnosis, and Gene Variants in Disease Heterogeneity

**DOI:** 10.3389/fped.2022.875877

**Published:** 2022-05-24

**Authors:** Heng Yang Lee, Azlin Ithnin, Raja Zahratul Azma, Ainoon Othman, Armindo Salvador, Fook Choe Cheah

**Affiliations:** ^1^Department of Paediatrics, Faculty of Medicine, Universiti Kebangsaan Malaysia Medical Centre, Cheras, Malaysia; ^2^Department of Pathology, Faculty of Medicine, Universiti Kebangsaan Malaysia Medical Centre, Cheras, Malaysia; ^3^Department of Medical Science II, Faculty of Medicine and Health Sciences, Universiti Sains Islam Malaysia, Nilai, Malaysia; ^4^CNC—Centre for Neuroscience Cell Biology, University of Coimbra, Coimbra, Portugal; ^5^Coimbra Chemistry Centre—Institute of Molecular Sciences (CQC-IMS), University of Coimbra, Coimbra, Portugal; ^6^Institute for Interdisciplinary Research, University of Coimbra, Coimbra, Portugal

**Keywords:** molecular screening, bilirubin, favism, hemolytic anemia, neonatal jaundice, peroxiredoxin 2, Heinz bodies, G6PD

## Abstract

Glucose-6-phosphate dehydrogenase (G6PD) deficiency is a prevalent condition worldwide and is caused by loss-of-function mutations in the G6PD gene. Individuals with deficiency are more susceptible to oxidative stress which leads to the classical, acute hemolytic anemia (favism). However, G6PD deficiency in newborn infants presents with an increased risk of hyperbilirubinemia, that may rapidly escalate to result in bilirubin induced neurologic dysfunction (BIND). Often with no overt signs of hemolysis, G6PD deficiency in the neonatal period appears to be different in the pathophysiology from favism. This review discusses and compares the mechanistic pathways involved in these two clinical presentations of this enzyme disorder. In contrast to the membrane disruption of red blood cells and Heinz bodies formation in favism, G6PD deficiency causing jaundice is perhaps attributed to the disruption of oxidant-antioxidant balance, impaired recycling of peroxiredoxin 2, thus affecting bilirubin clearance. Screening for G6PD deficiency and close monitoring of affected infants are important aspects in neonatal care to prevent kernicterus, a permanent and devastating neurological damage. WHO recommends screening for G6PD activity of all infants in countries with high prevalence of this deficiency. The traditional fluorescent spot test as a screening tool, although low in cost, misses a significant proportion of cases with moderate deficiency or the partially deficient, heterozygote females. Some newer and emerging laboratory tests and diagnostic methods will be discussed while developments in genomics and proteomics contribute to increasing studies that spatially profile genetic mutations within the protein structure that could predict their functional and structural effects. In this review, several known variants of G6PD are highlighted based on the location of the mutation and amino acid replacement. These could provide insights on why some variants may cause a higher degree of phenotypic severity compared to others. Further studies are needed to elucidate the predisposition of some variants toward certain clinical manifestations, particularly neonatal hyperbilirubinemia, and how some variants increase in severity when co-inherited with other blood- or bilirubin-related genetic disorders.

## Introduction

### Etiology

The glucose-6-phosphate dehydrogenase (G6PD) gene is approximately 18 kb in length and contains 13 exons and 12 introns. This gene is mapped onto the X chromosome at the Xq28 band and codes for the G6PD enzyme. G6PD deficiency is caused by loss-of-function mutations in the G6PD gene and follows an X-linked recessive inheritance pattern. These polymorphic variants usually have decreased enzyme activity and are often reported with reduced enzyme stability as well as disruptions in protein folding (1–3). Due to X-linked inheritance of the G6PD gene, males are more commonly affected compared to females. Males are either hemizygous wildtype (WT) or mutant for the G6PD gene, the latter of which results in G6PD deficiency. For females, there are two copies of the G6PD gene; having mutant alleles in both copies (homozygous) result in G6PD deficiency. Females having one WT and one mutant allele are heterozygous for the G6PD gene. Such individuals are categorized as partially deficient for G6PD. However, due to random X-inactivation, there may be a wide variation of G6PD levels in heterozygous females, and so some heterozygotes are effectively G6PD-deficient while some others may not be affected. Such variation presents a challenge in our effort to screen for G6PD deficiency, as partially deficient infants are often undetected with traditional conventional screening methods (4, 5).

### Epidemiology—Global and Local Insights

The global prevalence of G6PD deficiency was estimated to be 4.9% (6), amounting to approximately 400 million people affected worldwide, making it the most prevalent human enzyme deficiency. There is a wide geographical distribution of G6PD deficiency, with the highest prevalence reported across sub-Saharan Africa. This is followed by the Arabian Peninsula, central and southeast Asia, as well as in Mediterranean Europe and Latin America (6, 7). Due to high population density, the majority of G6PD-deficient individuals are predicted to be from Asian countries (7).

Since its discovery in 1956 by Carson et al. the prevalence and health burden contributed by G6PD deficiency have been determined in many parts of the world, made possible by the availability of cheap and rapid screening tests. In some countries neonatal screening programs are established as part of the strategy for prevention of severe neonatal jaundice. G6PD deficiency has been identified as a major risk factor in severe neonatal hyperbilirubinemia that may lead to the debilitating complication of kernicterus. The WHO initiated a working group to address G6PD deficiency as a significant global health issue with publication of a comprehensive classification of the disease, diagnosis, treatment, and prevention (8). A significant rate of severe jaundice causing kernicterus still plagues much of the world population especially in lower income economies. It is estimated that over 400,000 newborns are affected by jaundice each year, with approximately 75% of them residing in South-East Asia, Sub-Saharan Africa, and China (9, 10). The incidence of severe hyperbilirubinemia (over 340 mmol/L) is approximately 6.8% in Indonesia, with 2% of patients experiencing bilirubin-toxicity consequences (11).

Populations with medium to high prevalence rates may have a national newborn screening program for early detection of this condition and guidelines in close monitoring of affected infants. Early detection and intervention with phototherapy are simple cost-effective strategies to identify infants at-risk, monitor and treat hyperbilirubinemia expediently to prevent kernicterus. Although the diagnosis and preventative strategy may be rather straightforward, much is unclear about the pathophysiology and heterogeneity of this inherited genetic condition that underlies the severity of neonatal hyperbilirubinemia. The cost-effectiveness of such a healthcare strategy in countries with limited resources and a high disease burden with a rapid turnover of hospital bed occupancy needs due consideration. In countries such as Malaysia, G6PD deficiency is common and is an important cause of severe neonatal jaundice (12, 13). Mandatory screening of newborn infants has been implemented since 1986 by the Ministry of Health of Malaysia. Although the screening program has met with success, the lack of sensitivity of the recommended Fluorescent Spot Test (FST) remains an issue as it misses out on the diagnosis of some deficient female heterozygotes and G6PD-deficient individuals undergoing acute hemolysis. Additionally, not all G6PD-deficient neonates develop significant neonatal jaundice which raises the question whether certain variants are more at risk and if other coexisting risk factors play a role in influencing the severity of jaundice. In this respect, G6PD-deficient neonates monitored in the hospital during the first week of life that have an enzyme activity below 6.76 U/g Hb or the G6PD Kaiping (c.1388G > A) variant are associated with significant hyperbilirubinemia requiring phototherapy intervention (14). A quantitative enzyme activity test incorporating a panel of genetic variants at-risk for severe jaundice may help in target profiling and streamlining of care to affected infants. Yet another enigma is that, often in G6PD-deficient infants with severe hyperbilirubinemia, there may be no overt evidence of hemolysis in the peripheral blood film nor marked reticulocytosis (15), characteristic of most conditions causing hemolytic anemia and jaundice. Such findings indicate that perhaps a proportion of neonatal hyperbilirubinemia secondary to G6PD deficiency has a different mechanistic pathway other than an acute hemolytic crisis with exposure to oxidants, as seen in favism.

### Pathophysiology—Antioxidants and Gene Variants

**FIGURE 1:**
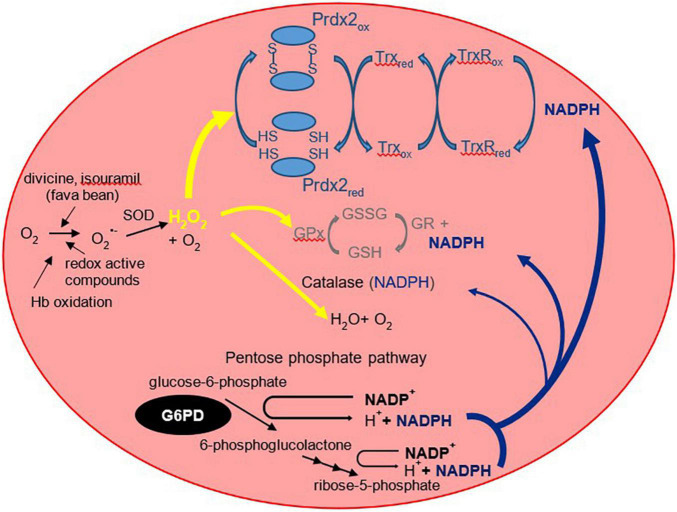
The three RBC antioxidant systems that are compromised in G6PD deficiency. The more familiar glutathione peroxidase/glutathione reductase (GPx/GR) and antioxidant mechanisms are shown in gray (middle) and the more recently recognized peroxiredoxin 2 system in light blue (top). Prdx2 is a thiol protein that is oxidized to an interchain disulfide then recycled predominantly by thioredoxin (Trx) and thioredoxin reductase [TrxR; (102)]. Prdx2, the third most abundant RBC protein, is present at a much higher concentration than glutathione peroxidase or catalase. It is highly reactive with peroxides and is favored to consume most of the intracellular H_2_O_2_ (17). NADPH is required as a reducing substrate for both GR and TrxR, and it protects catalase against inactivation. It is produced via the pentose phosphate pathway of which the first step is catalyzed by G6PD. By restricting the supply of NADPH, G6PD deficiency compromises the ability of all three antioxidant systems to detoxify hydrogen peroxide. O_2_^–^, superoxide; H_2_O_2_, hydrogen peroxide; GSH, reduced glutathione; GSSG, glutathione disulfide; SOD, superoxide dismutase. The “red” and “ox” subscripts refer, respectively, to the reduced and oxidized forms of the proteins.

The G6PD enzyme is constitutively expressed in cells and plays an essential role in the pentose phosphate pathway. It catalyzes the oxidation of glucose-6-phosphate into 6-phosphogluconolactone. Importantly, G6PD regenerates the reduced form of nicotinamide adenine dinucleotide phosphate [NADPH; (16)]. NADPH acts as a cofactor and reducing agent for enzymes such as glutathione reductase and thioredoxin reductase, catalyzing the reduction of glutathione (GSH) and thioredoxin (Trx), respectively (Figure 1). In turn, reduced Trx participates in the reduction of peroxiredoxins, which, together with GSH, form the main antioxidants of the red blood cell [RBC; (17, 18)]. These antioxidants reduce hydrogen peroxide and in doing so protect the RBC against oxidative damage. Perhaps the two more recognized clinical manifestations in G6PD deficiency are blackwater fever and favism, syndromes consequent to acute and massive hemolysis. As described by Luzzatto and Arese (19), hemolysis of G6PD-deficient RBCs in favism arises because the cells cannot generate sufficient NADPH to detoxify excessive hydrogen peroxide and other reactive oxidants. However, the traditional view that NADPH is required strictly for the glutathione peroxidase and catalase systems need to be revised with new insights in the form of another major player in RBC antioxidant defense, peroxiredoxin 2 (Prdx2).

Prdx2 is much more abundant than glutathione peroxidase and catalase. It is highly reactive and should therefore be the main consumer of hydrogen peroxide in RBCs at low to moderate oxidative stress. The antioxidant function of Prdx2 also depends on NADPH. Oxidized Prdx2 forms an interchain disulfide, which is recycled by thioredoxin and thioredoxin reductase, and this requires reducing equivalents from NADPH. The antioxidant activity of the Prdx system should be compromised when NADPH cannot be supplied by the pentose phosphate pathway. This was evidenced by RBC Prdx2 in G6PD-deficient infants, with a higher proportion shown to maintain the oxidized state and are poorly reactivated after peroxide challenge (20). Further, recent studies into the modeling of Prdx2 recycling after a hydrogen peroxide bolus implicated the role of various genetic variants of G6PD in Prdx2 reactivation. The different variants resulted in different rates in kinetic reactions that may affect Prdx2 recycling. Table 1 shows previously reported kinetic parameters of G6PD activity attributed to different variants (21, 22).

**TABLE 1:** Kinetic parameters in G6PD enzyme activity between some genetic variants against G6PD B (normal).

G6PD Gene	Kinetic parameter			
	G6PD activity (% of normal)	*K*_*M*_,*_*G*_*_6_*_*P*_* (μM)	*K*_*M*_,*_*NADP*_* + (μM)	*K*_*I*_,*_*NADPH*_* (μM)
B	100	38	6.5	7.1
Viangchan	3	105	12	19
Kaiping	3.8	40	3	*
Mahidol	17.2	40	*	*
Canton	14	28	*	*
Gaohe	12	31.5	*	*

*Indicates data that are not available, and the values are assumed similar to G6PD B. Table adapted from Beutler (21) and Coelho et al. (22).

Thus, a diminished activity of the Prdx2 antioxidant system may be a contributor to G6PD-related diseases, and this could also be dependent on the G6PD genetic variant. More research is needed to study the role of Prdx2 in jaundiced G6PD-deficient infants with and without acute hemolysis. The oxygen-carrying function of the RBC results in the generation of oxygen radicals from cellular metabolism, leading to constant exposure to endogenous and exogenous sources of oxidative stress. As such, the maintenance of GSH and Prdx2 antioxidants are essential to protect the RBCs from these reactive oxygen species (ROS). It should also be noted that RBCs lack other NADPH-producing enzymes, and any loss of NADPH cannot be compensated by other pathways. Therefore, the effect of G6PD enzyme deficiency leading to NADPH insufficiency is particularly marked in the RBC. The decreased NADPH production results in insufficient reduction of oxidized glutathione disulfide (GSSG) and Prdx2, thus rendering the antioxidant system less effective (20). In comparison, glutathione may have a key protective action in preventing oxidation of sulfhydryl groups in cytoplasmic and membrane proteins, especially in resisting severe oxidative challenge; favism was reported in cases of glutathione reductase and glutathione synthetase deficiency, even though levels of G6PD, and therefore NADPH, were normal (23). RBCs lacking in G6PD enzymes are susceptible to oxidative stress, potentially resulting in hemolysis especially when challenged with oxidative agents. However, the clinical distinction between acute favism and severe neonatal hyperbilirubinemia may be explained by the possible differing roles and contribution from the various antioxidant components in the RBC. A potential area of research would be to study this aspect in preterm infants as they are recognized to have a generally lower antioxidant capacity in the background of G6PD deficiency.

## Classes of Mutation and Spectrum of Deficiency

G6PD deficiency is a heterogeneous condition with 217 mutations reported as of 2016. The first 186 mutations were described and listed in detail by Minucci et al. (24), with 31 new mutations added thereafter by Gómez-Manzo et al. (25). Amongst these mutations, 182 (83.9%) are single nucleotide substitutions, 19 (8.7%) are multiple nucleotide substitutions, 11 (5.1%) are deletions, and 5 (2.3%) are intronic mutations. Of note, none of the 11 deletion variants were frameshift mutations; in all cases the number of deleted nucleotides were multiples of three and in line with the open reading frame. Therefore, the reading frame of the gene, which adopts a triplet nature, is not affected downstream of the mutation. Furthermore, of the 201 variants with nucleotide substitutions, 200 were missense variants instead of nonsense variants, meaning that the mutations cause a change in amino acid rather than a premature termination of translation. Since then, many more novel variants were reported, either through case reports from symptomatic patients [c.1375C > G; (26)] or genetic screening [c.1118T > C; (27)]. Other studies involving genetic analyses also reported 2 and 7 novel mutations in Malaysia and Qatar, respectively (28, 29). Such investigative approaches not only help to accelerate discovery of novel variants for diagnosis but also aid in the study of disease susceptibility and expansion of the panel for future genetic testing.

The one and only nonsense mutation recorded so far was reported in a heterozygous patient of Filipino descent and was termed G6PD Georgia (c.1284C > A), encoding for a truncated protein (30). As nonsense mutations had never been reported before, it was postulated that nonsense mutations resulted in a complete loss of G6PD enzyme and this would be embryonic lethal. This was corroborated by observations of embryonic lethality in mouse and *Caenorhabditis elegans* models, with oxidative stress damage shown to be the underlying cause (31). The heterozygosity of this patient could perhaps explain how G6PD levels are compensated by the WT allele; inheritance of this mutant in hemizygotes is expected to cause embryonic lethality.

According to the World Health Organization (WHO), G6PD variants are categorized into 5 classes depending on the degree of enzyme deficiency and severity of hemolysis (8). This classification was initially proposed by Yoshida et al. (32) but has since been widely recognized as the current standard WHO classification. The most severe mutations generally fall into Class 1, characterized by severe enzyme deficiency (<10% of normal activity) with chronic non-spherocytic hemolytic anemia (CNSHA). Class 2 variants are indicated by severe enzyme deficiency (<10% of normal activity) without CNSHA while Class 3 variants are presented with moderate to mild enzyme deficiency (10–60% of normal activity). Variants that result in very mild or no enzyme deficiency (60–100% of normal activity) are listed in Class 4; Class 5 variants are characterized by an upwards of twofold increase in enzyme activity (>200% of normal activity).

It has been more than three decades since this classification was introduced. A revision is warranted as more evidence emerge relating genotype with phenotype, genetic variant classes and enzyme activity, and discovery of novel mutations. A recent population-based study on the genotype-phenotype relationship unraveled complexities in the classification that need to be addressed (33). The G6PD Quingyan (c.392G > T) variant fell into Classes 2 and 3, highlighting the difference in residual enzyme activities between individuals (33). The classification also does not take into account heterozygosity, which results in a wider spread of residual enzyme activity, as well as the effect of compounding mutations which has been shown to synergistically decrease the catalytic activity and/or protein stability (34). Pietrapertosa et al. (35) reported that enzymatic activity were poor predictors of clinical manifestations and that they could be more related to genotype instead. A significant proportion of moderate or partially deficient patients (Class 3) still developed severe hyperbilirubinemia that require prompt phototherapy intervention (14), showing that higher residual enzyme activity level does not confer more protection against hyperbilirubinemia. A global initiative to study the severity of neonatal jaundice and the variants most at risk for hyperbilirubinemia will steer the development of specific tests in this era of genomic and precision medicine.

## Clinical Manifestation

Most individuals with G6PD deficiency are often asymptomatic. However, this can easily change when they are exposed to oxidative stress. In the newborn period, these oxidative triggers are rarely encountered than in older children and adults ingesting fava beans, or medications that predispose them to acute hemolytic anemia. Even so, nursing mothers ingesting fava beans and the use of mothballs have been reported to trigger acute hemolytic anemia with jaundice in the neonatal period. More commonly in newborn infants, G6PD deficiency presents as rapid onset and unremitting neonatal jaundice, which if unrecognized early, remains as one of the leading causes of kernicterus or BIND (36, 37).

### Acute Hemolytic Anemia

Fava beans (*Vicia faba*) contain high concentrations of glucosides called vicine and convicine, which are hydrolyzed in our body to form divicine and isouramil, respectively. Divicine and isouramil are aglycones that are highly reactive and produce ROS in the blood. As NADPH is insufficient in G6PD-deficient RBCs, the increased ROS are not eliminated effectively and thus cause damage to the RBCs. The levels of aglycones produced in our body are known to be affected by multiple factors, and the occurrence of acute hemolytic anemia in G6PD-deficient patients is reported to be dose-dependent (19).

The severity of favism was compared between three distinct G6PD-deficient variants in the Palestinian Gaza community (38). The three variants in comparison were G6PD Cairo (c.404A > C), G6PD Mediterranean (c.563C > T) and G6PD A- (c.202G > A, c.376A > G), as described by Vulliamy et al. (39) and Sirdah et al. (40). G6PD Mediterranean and Cairo were categorized as Class 2 variants while G6PD A- is a Class 3 variant. Hemolytic anemia was reported to be significantly more severe with G6PD Mediterranean and Cairo compared to G6PD A-, indicating a direct positive correlation between enzyme deficiency and severity of clinical manifestations. Additionally, G6PD Cairo was also observed with persistent symptoms and delayed recovery from favism (38). Favism has been reported in the perinatal period with cases of hydrops fetalis (41) and a newborn infant with hemolytic anemia secondary to maternal ingestion of fava beans before delivery (42). There were similar reports during the nursing period (43) and among infants who were exclusively breast-fed (44). In one of these cases, the mother reported eating fava beans 4 days prior to neonatal favism onset, which suggests that oxidative compounds derived from fava beans could be present in the breastmilk.

Severe neonatal jaundice from acute hemolysis can also arise from a G6PD-deficient infant’s exposure to environmental oxidative compounds. One example is mothballs, a common and cheap household item used as an insecticide when storing clothing articles in many Asian, Latin America and African communities. Mothballs contain naphthalene, a polycyclic aromatic hydrocarbon, which enhances the production of ROS, leading to oxidative stress in G6PD-deficient RBCs (45, 46). One of the more potent naphthalene-derived metabolites is alpha-naphthol, which causes hemolysis and severe anemia as well as Heinz bodies formation after accidental ingestion (47). Moreover, a 4-day old Panamanian male infant with G6PD deficiency developed severe jaundice and kernicterus only from direct contact with or inhalation of vapor from naphthalene-impregnated clothes, and subsequent genotyping revealed an underlying G6PD Mediterranean variant (48). Such potential severe consequences have led to calls for the ban of such substances in Australia (49). The use of henna as a traditional cosmetic agent in some cultures may also cause hemolysis. The leaves of the henna plant contain a potent oxidant, lawsone (2-hydroxy-1, 4-naphthoquinone), that was recently reported to cause acute hemolytic anemia and hyperbilirubinemia in a 4-day old infant (50).

In cases of breastmilk transmitted favism and naphthalene contact or vapor inhalation, the G6PD-deficient infants with acute hemolysis showed the presence of Heinz bodies in their blood films (44, 51). This was not the case in the massive acute hemolysis of indeterminate precipitating factors reported in a set of preterm Asian twins. The first twin succumbed to rapid deterioration from severe anemia and the second twin required exchange transfusions but, in both cases, there were no Heinz bodies in the blood film or hemoglobin in the urine (52). The peculiarity of this variable presentation is further discussed in the following section.

The other triggers of acute hemolytic anemia include certain medications such as anti-malarials, and to a lesser extent, bacterial or viral infection. Incidentally, it was the investigation into primaquine-sensitive RBCs that led to the discovery of G6PD deficiency (53). Primaquine and Pamaquine are antimalarial drugs known to induce hemolysis in G6PD-deficient patients. Other drugs that exert similar effects include Nitrofurantoin, Sulfones, and Sulfonamides such as Sulfanilamide and Sulfacetamide (54, 55). These drugs are either oxidative in nature or can generate oxidant compounds in the body, inducing oxidative stress to the RBCs leading to hemolysis. With infections, several reports have implicated the role of bacterial pneumonia and typhoid fever for the onset of hemolysis (56–58). Hepatitis viruses A and E, and cytomegalovirus have also been implicated in G6PD-deficient patients (59, 60). Although the specific mechanism of hemolysis induced by bacterial and viral infection is largely unclear, it has been postulated that phagocytic leukocytes may play a role, through generation of hydrogen peroxide during the phagocytic process (61).

### Neonatal Jaundice and Hyperbilirubinemia

**FIGURE 2:**
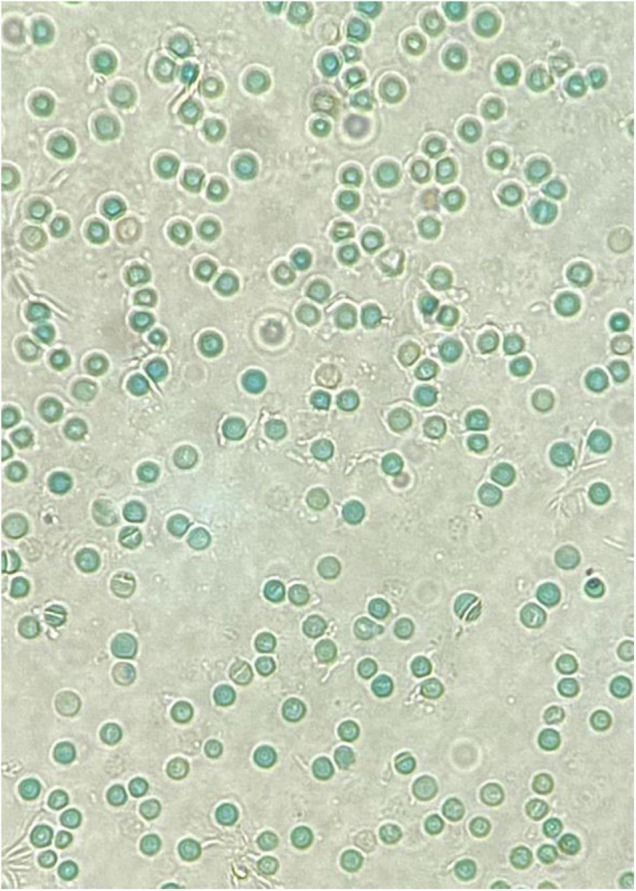
A crystal violet supravital staining of a peripheral blood film, viewed at 40x magnification. Note the absence of Heinz bodies in this blood film from a G6PD-deficient neonate with hyperbilirubinemia.

A major risk factor for neonatal hyperbilirubinemia is G6PD deficiency. Multiple reports have shown that G6PD-deficient infants are significantly predisposed to neonatal jaundice (62–64) and that it is more likely to be severe enough to cause kernicterus. Table 2 summarizes some of the variants reported to be associated with neonatal hyperbilirubinemia. Interestingly, in G6PD-deficient infants these conditions are poorly correlated with hemolysis and the presence of Heinz bodies. This could be because neonatal hyperbilirubinemia is not a result of acute hemolytic anemia, but rather through multiple endogenous factors in an infant’s body leading to susceptibility of jaundice (65). As an example, the crystal violet or supravital blood stain of an infant with significant hyperbilirubinemia who was G6PD-deficient did not show the presence of Heinz bodies (Figure 2). In contrast, the blood film of G6PD-deficient infants who developed hemolytic anemia after exposure to fava beans or naphthalene, as described earlier, had presence of Heinz bodies. Khefacha et al. (50) reported an *in vitro* formation of Heinz bodies in the RBC when henna was added directly onto a G6PD-deficient newborn blood sample. It would be useful if the genotype and peripheral blood film of this infant were available, as these may provide some insights on the *in vivo* clearance or the existence of these Heinz bodies.

**TABLE 2:** Specific G6PD variants that have been reported to be associated with neonatal hyperbilirubinemia.

G6PD variant	cDNA nucleotide substitution	Amino acid substitution	Mutation class	References
Nashville, Anaheim, Portici	1178G > A	Arg393His	I	(95)
Campinas	1463G > T	Gly488Val	I	(95)
Harilaou	648T > G	Phe216Leu	I	(95)
Volendam	514C > T	Pro172Ser	I	(95)
Canton, Taiwan-Hakka, Gifu-like, Agrigento-like	1376G > T	Arg459Leu	II	(14)
Kaiping, Anant, Dhon, Sapporo-like, Wosera	1388G > A	Arg463His	II	(14)
Gaohe	95A > G	His32Arg	II	(14)
Viangchan, Jammu	871G > A	Val291Met	III	(14, 96)
Mahidol	487G > A	Gly163Ser	III	(14, 96)
Mediterranean, Dallas, Panama, Sassari	563C > T	Ser188Phe	II	(14, 96–98)
Orissa	131C > G	Ala44Gly	III	(99)
A^–202A/376G^	202G > A 376A > G	Val68Met, Asn126Asp	III	(96)
A^–376G/968C^, Betica, Selma, Guantanamo	376A > G 968T > C	Asn126Asp, Leu323Pro	III	(96)
Union, Maewo, Chinese-2, Kalo	1360C > T	Arg454Cys	II	(96)
Akrokorinthos	463C > G	His155Asp	II-III	(96)
Belem	409C > T	Leu137Phe	II	(96)
Santamaria	376A > G 542A > T	Asn126Asp, Asp181Val	II	(96)
Hamburg	827C > T	Pro276Leu	I	(100)
Herlev	592C > A	Arg198Ser	I-II	(101)

Furthermore, neonates have larger sized RBCs, and these cells have a shorter lifespan compared to that of the adult (120 days). The lifespan of the neonatal RBC ranges between 60 and 90 days, while that of preterm infants are even shorter at 35–50 days (66). The neonatal RBC has overall lower enzyme levels of glutathione peroxidase and carbonic anhydrase, which are important to regulate cell homeostasis and membrane integrity; such factors may increase susceptibility to oxidative damage (67). A G6PD-deficient state may accentuate the decreased life span of the RBC. Together, these result in increased bilirubin load in the blood circulation and in liver cells of an infant. Moreover, the infant’s liver is relatively functionally immature, resulting in a decreased uptake of bilirubin from the blood. One reason for this is the low concentration of ligandin in the infant’s liver cells. This protein is responsible for binding to bilirubin in the liver, and it only increases in concentration during the first few weeks of life (68). The other reason is due to decreased uridine diphosphate glucuronosyltransferase activity in liver cells which results in decreased bilirubin conjugation in the liver. It was also reported that co-inheritance of a uridine diphosphate glucuronosyltransferase 1A1 (UGT1A1) gene variant is an added risk factor for neonatal hyperbilirubinemia in G6PD-deficient male neonates (69). Altogether, an elevated bilirubin level in the blood as well as ineffective clearance from the liver causes the build-up of serum bilirubin leading to neonatal hyperbilirubinemia, which occurs more frequently and severely in G6PD-deficient infants (65).

Another distinctive feature of the neonatal RBC is the presence of the predominant fetal hemoglobin (HbF). HbF has a tendency to denature with oxidative stress and precipitate to disrupt the RBC membrane, contributing to the shortened RBC lifespan. These denatured precipitated HbFs form the Heinz bodies and, as such, most hyperbilirubinemic infants secondary to G6PD deficiency should display characteristics of Heinz bodies. However, this is not the case. A possible explanation is that there is a relatively more efficient clearance of Heinz bodies during the transition period of fetal to adult Hb. Additionally, there is an alternative pathway that reduces the susceptibility of the Hb to oxidative denaturation. A significant fraction of the heme moeity may be converted to metabolites other than bilirubin, such as mesobilifuscins, that are excreted in the bile/feces and urine (70). More research is needed to explore the link between the different genetic variants of G6PD and this alternate pathway in Heinz body catabolism that could possibly explain this hematological peculiarity.

**FIGURE 3:**
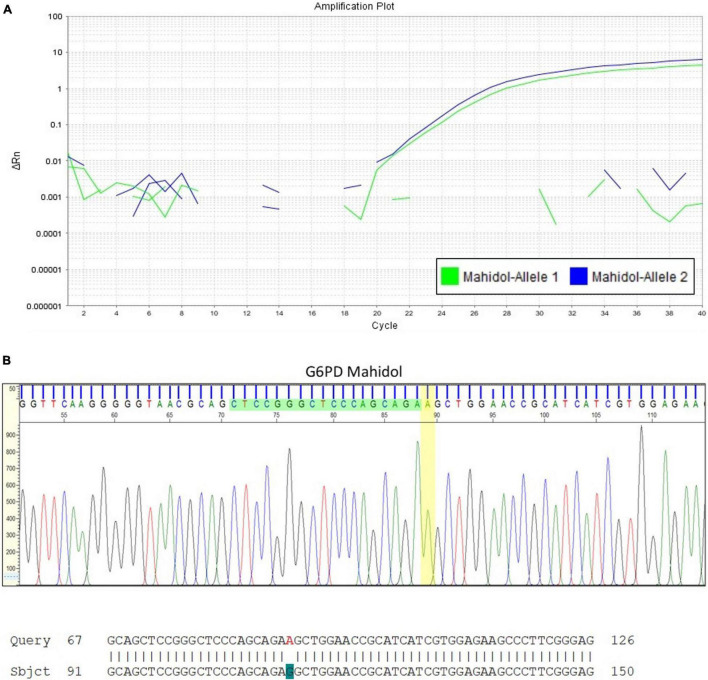
Quantitative Polymerase Chain Reaction (qPCR) for G6PD variant analysis in a newborn infant with early onset jaundice. **(A)** This assay confirmed that the female infant with severe hyperbilirubinemia at day 3 of life but with G6PD enzyme activity reported within the normal reference range, was heterozygous for G6PD Mahidol (c.487G > A). DNA sequencing result is shown in **(B)**.

Heterozygote females are often considered only as carriers, leading to under-diagnosis of G6PD deficiency as the underlying cause of severe hyperbilirubinemia. A case to highlight here is a Malay baby girl who was born at term and developed jaundice at day 3 of life. Basic hematological indices were normal except for the reticulocyte count that was increased at 6.28%. The peripheral blood smear findings showed no evidence of hemolytic anemia. G6PD enzyme level taken on admission was 7.47 U/g Hb (normal, above 6.76 U/g Hb). The diagnosis of G6PD deficiency could be easily dismissed if not for the understanding that the enzyme activity was spuriously elevated due to the increased reticulocyte count. Further investigation with molecular analysis for G6PD gene mutations revealed that the patient was heterozygous for G6PD Mahidol (c.487G > A; Figure 3).

## Laboratory Tests and Diagnostic Methods

The emergence of point of care testing has increased the opportunity for screening this globally prevalent genetic disorder. A simple, rapid, and accurate point-of-care test (POCT) made accessible to resource limited parts of the world with high prevalence of G6PD deficiency may help in early diagnosis and to curb severe hyperbilirubinemia with counseling and advice on taking the necessary steps for prompt intervention. Molecular screening for gene variants may not appear practical or cost-effective traditionally because of the infrastructure and technical expertise required, but advancements are progressively making this more widely accessible in the form of gene chips.

### Fluorescent Spot Test

G6PD status is usually determined by measuring enzyme activity from whole red blood cells. Screening of cord blood in neonates for G6PD deficiency is a popular method especially in low-middle income countries with high prevalence. Individuals who live in malaria endemic areas are also screened for G6PD status before administration of 8-aminoquinolone compounds. The most widely used G6PD deficiency screening method is the fluorescent spot test (FST), a semi-quantitative assay. Although inexpensive and easy to carry out, this method is only able to detect cases with 20% or less of mean normal G6PD activity and misses most of the moderate G6PD activity especially in female heterozygotes (4).

### Quantitative Glucose-6-Phosphate Dehydrogenase Activity

Several quantitative test kits have been introduced in recent years which are superior to the FST in terms of accuracy and sensitivity. The OSMMR2000-D G6PD assay kit (R&D Diagnostics Ltd., Aghia Paraskevi, Greece) is one such tool that is available in the market. The G6PD activity is quantified relative to a control sample and is normalized for hemoglobin concentration using a spectrophotometer. Absolute values of G6PD activity can vary between different assays and hence results need to be translated to the population, relative to the male population-specific adjusted mean (71). The overall mean values for G6PD activity have to be optimized in individual laboratories to determine the cut-off points for moderate and severe G6PD deficiency. As defined by the WHO, upper and lower limit cut-off points for moderate deficiency are set at 60 and 10% of the normal mean, whilst 10% and below of normal mean G6PD activity is classified as severe deficiency. The calculation of normal mean has to be performed and updated with every fresh batch of reagents to account for the deviation in the control sample. A point to highlight is that the normal reference range needs to be interpreted with caution in cases of hemolysis, reticulocytosis, and for preterm infants, as these are associated with elevated enzyme levels.

### Point-of-Care Testing

Alternatively, the CareStart™ G6PD Biosensor (Access Bio, Somerset, New Jersey, United States) is a handheld digital device measuring total G6PD activity through electrochemical quantification of NADPH production. This device is preferred in certain situations due to its practicality and convenience in producing a rapid, bed-side result readout. This point-of-care test (POCT) kit is able to incorporate both enzyme activity and hemoglobin concentration measurement into the result, which is automatically calculated within the device. The CareStart™ G6PD Biosensor 1 (Wells Bio, Seoul, Republic of Korea), a newer generation and more quantitative method of testing, is also reportedly more sensitive (72). The Hb and reticulocyte count in this method are measured separately in an automated blood cell counter and the enzyme activity is then expressed in U/g Hb, making it less of a POCT.

Due to its simplicity, the CareStart™ G6PD Biosensor POCT becomes an increasingly validated test for use in screening of newborn infants for G6PD deficiency. Analysis of 216 newborn infants against the FST and G6PD enzyme activity assay showed intraclass correlation coefficient of 0.90, with 100% sensitivity and 96% specificity (73). In a similar study among Chinese neonates, this test showed a sensitivity of almost 100% in deficient cases involving both males and females with residual enzyme activity below 60% (74). A quantitative POCT on a digital microfluidics platform was recently reported (75). As digital microfluidic technology compacts all steps from sample processing to analysis and waste handling in a disposable cartridge, this fully automated system is an attractive POCT for smaller hospital use when laboratory facilities and trained staff are not available or limited. A cartridge can be loaded with up to six patient samples. The preliminary results were promising, showing a strong correlation with the standard G6PD enzyme activity assay (75).

### Molecular Screening of Glucose-6-Phosphate Dehydrogenase

Molecular techniques remain the most unambiguous method in the diagnosis of G6PD deficiency. The G6PD Deficiency GenoArray Diagnostic Kit (Hybribio, Sheung Wan, Hong Kong, China) is an all-in-one integrated polymerase chain reaction (PCR), hybridization, and gene chip processing system based on flow-through hybridization technology which can provide a rapid and accurate nucleic acid analysis of a patient sample. This test kit can detect up to 14 G6PD gene mutations in one run and the results can be directly visualized as a precipitated dot on the membrane template to provide a readout of the patient’s G6PD allele. The mutation analyses could be interpreted as heterozygous, homozygous or compound heterozygous without additional PCR runs since the wild type probes have been incorporated into the test kit for comparison. However, as the selection of G6PD mutations for the test kit may be limited to the common mutations known to occur in that locality, further sequencing is necessary if rarer or novel mutations are suspected. Such molecular techniques have been applied on a large scale and population-based studies, availing genetic counseling for families presenting with different variants (33, 76). Application of molecular techniques for diagnostic purposes could be implemented with an established G6PD mutation database (77, 78). The flexibility of this array kit allows customization in the number and the preferred variants onto the membrane template, suited to the local requirements (28).

## Glucose-6-Phosphate Dehydrogenase Activity in Relation to Gene Mutation

### Position of Mutation

Biochemically, there is a huge correlation between the position of mutation and the severity of deficiency. Using three specific examples reported by Beutler et al. (79), the mutants G6PD Calvo Mackenna (c.1138A > G), G6PD Riley (c.1139T > C), and G6PD Wisconsin (c.1177C > G) were reported in patients presenting with CNSHA. All three mutants are categorized under Class 1 variants and these mutations were found in exon 10 of the G6PD gene. Many other mutations that result in CNSHA are clustered in exon 10 as well. Some examples of these are G6PD Tennessee (c.1465C > G), G6PD Veracruz (c.1094G > A), and G6PD Yucatan (c.1285A > G), as reported by Vaca et al. (80) and McDade et al. (81). Functionally, exon 10 encodes for part of the G6PD enzyme that makes up the dimerization interface and structural NADP^+^ binding interface (82–84). Similarly, the variants G6PD Kaiping (c.1388G > A) and G6PD Canton (c.1376G > T), common among those of Chinese ancestry are reportedly more at risk for severe hyperbilirubinemia. The proximity of these two mutations to the putative NADP-binding site of G6PD may contribute to the more severe form of disease (85). Crystal structure analysis and molecular weight calculation has shown that the native G6PD enzyme exists predominantly in its tetrameric form, while the formation of a homodimer is crucial for its catalytic function (83, 86). Additionally, the inclusion of a structural NADP^+^ molecule in each subunit of G6PD is vital as it contributes to the stability of these multimers (83, 84). Au et al. (83), using the first crystal structure of G6PD, showed that the substitution of Arg with Leu at a highly conserved amino acid area near to the NADP^+^ binding site in G6PD Canton affects the stability of the molecule. Together, these findings indicate that exon 10 is an important domain to maintain G6PD structural integrity and functionality, and mutations in this region of the enzyme commonly result in the most severe form of deficiency.

Outside of exon 10, some other examples of mutations that lead to CNSHA include G6PD Zacatecas (c.770G > T) and G6PD Palermo (c.769C > A, c.770G > T), reported by Vaca et al. (80) and Rigano et al. (87). Both mutations are in exon 7 of the G6PD gene. Through X-ray structure analysis, the mutations were found to be in proximity of the G6PD active site and were postulated to affect the substrate binding interface (25). Additionally, G6PD Zacatecas causes a change of amino acid from Arg to Leu (p.R257L) and this disrupts the salt bridge between R257 and E473 (88). Overall, the position of different mutations can affect protein catalytic activity and stability to various degrees, with mutations in specific region of the protein leading to more severe deficiency.

### Amino Acid Replacement

One other aspect of correlating mutations and their corresponding disease severity is through the analysis of amino acid replacement. The basis is that different mutations affect the functional and structural parameters of a protein differently, depending on the similarity or dissimilarity between the WT and the replacing mutant amino acid. The calculation of similarity is based upon variables such as amino acid charge and size, as well as the physico-chemical distance (89). Grantham (90) introduced the Grantham Difference which scores pairs of amino acids based on the difference in their atomic composition, polarity, and volume.

As an example, G6PD Mahidol (c.487G > A) and G6PD Plymouth (c.488G > A) are variants that affect the same amino acid, G163 (91, 92). G6PD Mahidol is a Class 3 variant causing a serine substitution (G163S) and G6PD Plymouth is a Class 1 variant with aspartic acid substitution (G163D). While both amino acid replacements have side chains that are polar as opposed to the original glycine, aspartic acid also introduces a negative charge to the surrounding, as well as being larger than serine. Consequently, Huang et al. (92) reported that while both variants were significantly less stable compared to WT, G6PD Plymouth was more severely affected than G6PD Mahidol. Both variants had similar kinetic parameters as the WT, indicating that though catalytically unchanged, the stability of these variants is markedly reduced, with G6PD Plymouth present in even lower amounts and ultimately leading to more severe clinical manifestations. Of interest, in our previously published study, neonates with the G6PD Gaohe (c.95A > G) variant had severe hyperbilirubinemia and were five times more likely to require phototherapy during the first week (14). The substitution of the amino acid (His to Arg) may alter the charged amino acid configuration, which could affect the enzyme activity more than neutral or hydrophobic amino acids (93).

In general, G6PD mutations are position-specific and may cause severe deficiency if they abrogate functional domains of the protein, such as dimerization and alteration of the substrate binding interface. Besides, G6PD mutations can also affect stability of the protein, through changes of its properties such as charged interactions, hydrophobicity and steric hindrance. Resultantly, the protein could be misfolded or subjected to erratic post-translational modification, ultimately being more susceptible to proteolysis and other forms of degradation in the RBC (88).

## Summary

G6PD deficiency is the most common enzyme deficiency known to affect humans. G6PD deficiency in RBCs results in clinical manifestations stemming from the inability of G6PD-deficient RBCs to deal with ROS. In infants, the risk of neonatal jaundice and hyperbilirubinemia is increased in frequency and severity (65). G6PD deficiency in newborn infants is well-recognized to cause a sudden rise in bilirubin levels that in many cases only exchange transfusion may be able to rapidly alleviate the toxicity from bilirubin accumulation. The impact of this deficiency on neonatal health especially in hyperbilirubinemia and kernicterus has led many countries with moderate to high prevalence to implement universal screening. Even so, much more can be done in newborn screening globally. Many countries in the developed world are still stratifying according to ethnicity risk as a pre-requisite for screening. With migration and inter-marriages, this may no longer be quite applicable.

It is plausible that hyperbilirubinemia and acute favism may be two separate entities mechanistically. The former, often lacking in overt hemolysis and Heinz bodies, may be more of a bilirubin accumulation and clearance problem. The role of antioxidants such as Prdx2 in RBCs are only beginning to unravel. The predisposition of different variants enhancing the susceptibility of oxidizing denatured Hb (Heinz bodies) to metabolites other than bilirubin is an area that needs to be explored to gain new insights on the heterogeneity of this disease phenotype. Acute favism, on the other hand, is a classic case of RBC membrane disruption. Stronger oxidants could be involved, overwhelming the glutathione system that may be primarily involved in preventing oxidation of protein sulfhydryl groups in the RBC membrane from lysis.

The discovery of G6PD crystal structures and subsequent analyses provided us with further insights into this enzyme deficiency, since specific mutations can now be plotted on a 3D position in the protein structure. This information enables us to elucidate the effects of individual mutations and provide us with the genotype-phenotype correlation (2). The percentage of residual enzyme activity based on WHO classification is not necessarily predictive of the clinical phenotype and risk of severe disease. Rather, mutations affecting the dimerization interfaces of the G6PD protein and in proximity to the NADP^+^ binding site may cause more detrimental effects compared to mutations of residues in other less conserved sites. Mutations could also lead to G6PD instability and disrupts protein folding which affects the amount of functional protein *in vivo*. Further studies should investigate the differences and to distinguish between the G6PD variants, their combinations, and co-inheritance with other genes for bilirubin-related metabolism in favism and severe hyperbilirubinemia. It is timely that a WHO working committee is regrouped to review the G6PD classification, stratifying of risks, diagnosis, therapy, and prevention, focusing on neonatal hyperbilirubinemia.

The development of affordable and accurate quantitative enzyme POCTs will enable greater opportunities for a more extensive coverage in newborn screening for G6PD deficiency. Advances in molecular techniques have led to the determination of the G6PD gene abnormalities that are more common in various parts of the world. The molecular profile has also provided some insights into the original gene pool of some of the local ethnic groups. These databases could be useful for improved detection, diagnosis, and more cost-effective strategies in managing G6PD deficiency in affected newborn infants.

Further research is emerging to characterize different variants with co-existing gene polymorphisms to relate to the bilirubin metabolism pathway and reactions to various oxidants. In this era of precision and personalized medicine there is an increasing need to develop a POCT with molecular genetic testing that produces a risk assessment based on a comprehensive profile for targeted therapy. Perhaps in addressing G6PD deficiency, the most promising in the horizon lies in drug development designed to correct the structural defects caused by the various genetic mutations that are becoming increasingly clearer (94).

## Author Contributions

FC: conceptualization. HL, AI, RA, AO, FC, and AS: research and manuscript writing. HL and FC: review and editing. All authors contributed to the article and approved the submitted version.

## Conflict of Interest

The authors declare that the research was conducted in the absence of any commercial or financial relationships that could be construed as a potential conflict of interest.

## Publisher’s Note

All claims expressed in this article are solely those of the authors and do not necessarily represent those of their affiliated organizations, or those of the publisher, the editors and the reviewers. Any product that may be evaluated in this article, or claim that may be made by its manufacturer, is not guaranteed or endorsed by the publisher.
